# Resident- and family-led huddles for collaborative care planning in long-term care: a feasibility study

**DOI:** 10.1093/geroni/igaf116

**Published:** 2025-10-23

**Authors:** Lisa A Cranley, Linda McGillis Hall, Wendy Duggleby, Shoshana Helfenbaum, Daniel Galessiere, Raquel M Meyer, Gajan Sivakumaran, Danielle Just, Lauren MacEachern, Katherine S McGilton

**Affiliations:** Lawrence Bloomberg Faculty of Nursing, University of Toronto, Toronto, Ontario, Canada; Lawrence Bloomberg Faculty of Nursing, University of Toronto, Toronto, Ontario, Canada; Faculty of Nursing, University of Alberta, Edmonton, Alberta, Canada; Ontario Centres for Learning, Research and Innovation in Long-Term Care at Baycrest Academy for Research and Education, Toronto, Ontario, Canada; SE Health (Saint Elizabeth Health Care), Markham, Ontario, Canada; Pallium Canada, Ottawa, Ontario, Canada; School of Medicine, Queen’s University, Kingston, Ontario, Canada; Ontario Health, Toronto, Ontario, Canada; Davis Pier, Toronto, Ontario, Canada; Lawrence Bloomberg Faculty of Nursing, University of Toronto, Toronto, Ontario, Canada; KITE Research Institute, Toronto Rehabilitation Institute-University Health Network, University of Toronto, Toronto, Ontario, Canada

**Keywords:** Communication, Family engagement, Advocacy

## Abstract

**Background and Objectives:**

Engaging long-term care residents and families in decisions and as active partners in care can improve the quality of care. However, barriers to effective communication among the care team, residents, and families remain. This study aimed to assess the feasibility, acceptability, and satisfaction with an intervention to engage residents, their family, and team members in a collaborative approach to care planning to support person- and relationship-centered care.

**Research Design and Methods:**

A multi-method approach was utilized. The intervention included: leadership coaching sessions; an education session with team members and leaders for communication skills training; and resident- and family-led care planning conversations (huddles) that included huddle training.

**Results:**

The intervention was found to be feasible to implement and was acceptable to participants. The leadership coaching sessions and the huddles could be feasibly conducted in-person or virtually. Participants were satisfied with the leadership coaching and the huddles, and the communication tool was found to be useful. Suggestions were made to further innovate huddles.

**Discussion and Implications:**

Huddles provided a structured approach and relational process that facilitated communication among team members, residents, and families. The communication tool provided a common language for discussing resident care. The leadership coaching sessions and huddles were feasible to implement and can be adapted for virtual delivery. Huddles are a promising practice for engaging residents and families in care planning, but further testing is needed.

**Clinical Trial Registration:**

NCT04026698

Innovation and Translational Significance: Partnering with residents of long-term care homes and their families in care planning decisions can enhance the quality of care. However, this collaboration can be hindered by ineffective communication among the care team, residents, and families. We assessed the feasibility, acceptability, and satisfaction of a multicomponent intervention aimed at improving communication and collaboration during care planning. The intervention featured resident- and family-led huddles, which introduced an innovative approach to care planning in long-term care homes. Huddles were feasible to implement and perceived as acceptable by participants. Huddles are adaptable for virtual delivery, expanding their potential application for long-distance caregivers.

## Background and objectives

Canada’s population aged 85 and older is one of the fastest-growing age groups.^1^ By 2050, the number of Canadians in this age group is expected to exceed 2.7 million,^1^ with over 1.2 million residing in Ontario.^2^ Long-term care (LTC) requirements are projected to increase by approximately 38% in the next 10 years.^3^ A Canadian report highlighted the long-standing underinvestment in the LTC sector, leading to deficiencies in staffing levels, quality and safety, wages, funding, and oversight.^4^ Recognizing the need to address these gaps has led to federal investments in LTC, and federal and provincial initiatives; for example, Canada recently launched its first national Dementia Strategy,^5^ and the Fixing the LTC Act in Ontario in 2021.^6^ Additionally, the Safe LTC Act is proposed federal legislation currently under development to address quality, safety, and availability of LTC services.^7^ These initiatives acknowledge the crucial role family caregivers play as members of the care team in enhancing a resident’s well-being and quality of life,^5-7^ and emphasize the need to improve support for these caregivers.^5^

Indeed, engaging residents and families as active partners in care decisions can improve residents’ quality of care.^8-10^ In a recent literature review, 3 main factors enabled residents’ and families’ engagement with decisions about their care: (a) a positive culture of collaborative relationships; (b) willingness to engage; and (c) communicating with intent to share and support.^11^ However, challenges in communication and relationship-building between teams and families have been identified, including ineffective communication channels^12^ and insufficient information provided to families.^9^ These barriers can lead to misunderstandings, often influenced by factors such as staff turnover and limited time during shifts.^9^ Our previous research found that communication between team members and family can often be reactive (eg, in response to a family concern), highlighting the need for more proactive communication strategies among team members, including involving personal support workers, and residents and family in care planning.^12^ Communication barriers between team members and residents can also arise from factors such as insufficient staff training in communication skills or residents experiencing difficulties due to cognitive and/or sensory impairments.^13^ Engaging residents and their families in care planning discussions with team members and incorporating the residents’ perspectives and needs can enhance communication and decision-making about care,^11^ supporting high-quality, safe, person- and relationship-centered care.^8^^,^^12^

Communication tools could help to support person-centered care and build partnered relationships among residents and families, and the care team. Communication tools have been developed to support interdisciplinary team communication (eg, TeamSTEPPS, SBAR: Situation, Background, Assessment, Recommendation).^14^ However, few communication tools exist to empower residents and families to participate in care planning.^15^ Education or training sessions and meetings (formal or informal) that involve team members, family, and residents could further optimize communication processes.^16^

There is a need for innovative and sustainable care models and practices that meaningfully engage residents and families to promote effective communication and collaborative care planning in LTC. Meaningful engagement of residents and family with lived experience as active partners in co-designed processes intended for them is considered best practice, as it optimizes the inclusion of their perspectives.^8^^,^^17^ Older adults with dementia who are engaged in meaningful activities experience positive outcomes such as improved well-being.^18^ Huddles are an intervention that could meaningfully engage residents and family to lead a collaborative approach to care planning and support proactive communication. Huddles are focused yet brief gatherings of functional groups (eg, interdisciplinary healthcare teams) that create time and space for information exchanges, two-way communication, and sharing of expectations.^19-21^ Resident- and family-led huddles are an innovation that could ensure that their voices are represented to optimize the inclusion of their perspectives and acknowledge them as active partners in care. A scoping review on huddle effectiveness for care quality found that 64% of included studies reported improvements in team efficiency and communication; studies also reported positive outcomes such as timely care.^19^ Huddles have been successfully implemented across a variety of healthcare settings to improve team processes and quality of care.^19^ Although their purposes may differ, huddles are commonly used to enhance team communication, identify issues that need resolution, and plan for quality improvement.^19^

Studies implementing huddles in LTC homes have reported improved team collaboration,^22^^,^^23^ increased team support,^22-24^ and better communication.^22^^,^^23^^,^^25^ Additionally, these studies have found improvements in team member job satisfaction^22^ and reductions in their overall moral distress.^24^ However, huddles have been less visible in LTC compared to other healthcare settings, and there remains a gap in incorporating various perspectives in huddles beyond the immediate team.^19^ Of the *n* = 158 studies included in the scoping review by Pimentel and colleagues (2021),^19^ only 7 were conducted in LTC settings for older adults. Four of the 158 studies involved patients and/or their families in huddles, which had also reported improvements in team efficiency and communication.^19^ Patients and/or their families participated in admission huddles to encourage active involvement in their care,^26^ Gemba whiteboard huddles to support practice changes,^27^ and post-fall huddles to discuss potential causes and strategies to prevent falls.^20^^,^^28^ However, in these 4 studies, patients and family members did not lead the huddles, nor were these conducted in LTC settings.

The aim of this study was to assess the feasibility, acceptability, and satisfaction of an intervention that engaged LTC home residents, their family caregivers/care partners, and the care team in a collaborative care planning approach to support person- and relationship-centered care. The intervention included 3 components: (1) leadership coaching for members of the leadership team to support the care team in huddles and in quality improvement; (2) an education session with team members and leaders for interprofessional communication and collaboration skills training; and (3) resident- and family-led huddles to facilitate collaborative care planning with the care team.

### Theoretical framework

Development of the intervention components was guided by the interprofessional shared decision-making model (IP-SDM).^29^^,^^30^ The IP-SDM model conceptualizes shared decision-making beyond the traditional physician-patient dyad, involving other healthcare providers, as well as family members, to support a more integrated and collaborative approach to decision-making.^29^^,^^30^ The model highlights the importance of actively involving the patient in the decision-making process as a member of the interprofessional healthcare team and recognizing their values and preferences.^29^^,^^30^ A health professional trained as a decision coach can support the patient’s involvement in decision-making.^29^^,^^30^ The IP-SDM model emphasizes communication and collaboration among all individuals involved in decision-making to foster shared knowledge, mutual trust, and understanding.^29^^,^^30^

The leadership coaching and education sessions integrated relational and goal-oriented strategies to support team communication and collaboration. The purpose of the huddles was for resident-family dyads to lead a 15-minute care conversation (same dyad for each huddle) focusing on one topic per huddle about the resident’s care. The huddle format was specifically designed to promote relational interactions that build trust and encourage an appreciative approach to care planning.^31^ The goal was to establish a process that fostered communication and collaboration among all participants, supporting person- and relationship-centered care. Similar to a decision coach, leaders were trained as huddle coaches to guide the huddle process and offer continuous support and encouragement to all participants, with an emphasis on empowering residents and family members as huddle leads.

## Research design and methods

**Figure 1.**
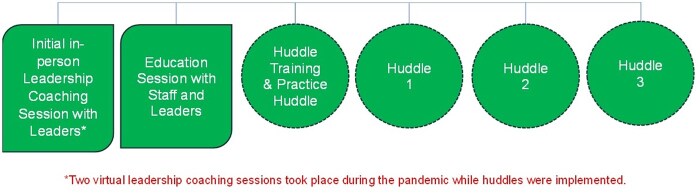
Intervention components.

A multi-method approach was used to evaluate the feasibility, participant acceptability, and satisfaction with a collaborative care planning intervention. The study was conducted in 2 phases. In phase 1, a communication tool was developed to empower and guide resident and family dyads to lead a care planning conversation (15-minute huddles) with the care team. Phase 2 involved implementation of the intervention (see Figure 1). This paper reports on our findings from Phase 2. We assessed the feasibility of the intervention implementation, as well as participant acceptability and satisfaction with the leadership coaching, huddles, and the communication tool developed for use in the huddles. Feasibility examines whether an intervention is practical and achievable (can it be done), and if so, identifies the best approach for its implementation.^32^ It also considers the resources necessary for delivering the intervention.^33^ When evaluating feasibility, we also considered the leadership coaching and huddle dose (amount, frequency, duration) and the context in which the intervention was implemented^33^ (eg, adapting its implementation during the COVID-19 pandemic). Acceptability assesses the appropriateness, suitability, and usefulness of the intervention, as perceived by those receiving the intervention.^33^ Participant satisfaction captures their perceptions of what aspects of the intervention they liked and disliked, as well as their suggestions for improvement.

### Setting and sample

The study took place in 4 accredited LTC homes in a large metropolitan city in Ontario, Canada. There are approximately 78 000 LTC residents across Ontario.^34^ Three of these LTC homes were for-profit (chain ownership), whereas one home was not-for-profit, and they ranged in size from 160 to over 300 beds. We purposefully recruited urban LTC homes to facilitate in-person delivery of the intervention. LTC homes were eligible if they had both a Resident Council and a Family Council to support the recruitment plan. Both for-profit and not-for-profit LTC home ownership models were eligible to enhance generalizability. In Ontario, 57% of LTC homes are owned by private for-profit organizations, 27% are owned by private not-for-profit organizations, and 16% are publicly owned.^35^ Secured dementia care units within LTC homes were excluded. Eligible participants included leadership team members (eg, directors of care, administrators, managers, educators, resident care coordinators), healthcare providers (eg, registered nurses, registered practical nurses, allied healthcare providers, personal support workers), and support staff (eg, activation aides, housekeepers/environmental service workers, dietary aides/food service workers). Healthcare providers and support staff (hereafter collectively referred to as team members) were eligible to participate if they worked full-time hours. Residents were eligible to participate if they could communicate verbally, were English-speaking, and did not have severe cognitive impairment. Family (relative, neighbor, or friend) were eligible if they were the resident’s substitute decision-maker and visited the resident at least monthly. We used convenience (quota) sampling, aiming to recruit 20 team members, 6-8 leaders, and 10-12 resident-family dyads total from 1-2 neighborhoods in each LTC home. The quota was set to achieve a participant group size that enabled simulation-based education activities. An honorarium was provided to each LTC home for team participant shift replacement.

Ethics approval was obtained from the University of Toronto Health Sciences Research Ethics Board (Project ID #36220) and our research team’s study partners’ organization (Project ID #18-32). Operational approval was obtained from participating LTC homes, and ethics approval as required by one study site (Project ID #1920). Informed consent was obtained from all participants. The Principal Investigator (PI) conducted information sessions for team members during team (staff) meetings and for residents and family during Resident Council and Family Council meetings to explain the study and invite them to participate. Managers or resident care coordinators at each study site also invited residents and family to participate in the study.

### Phase 1 communication tool development

Phase 1 (2019) involved the development of a paper communication tool for residents and family dyads to guide care conversations with teams through huddle discussions about an aspect of resident care.^15^ The communication tool is composed of a list of 5 questions structured around a modified SBAR communication framework: Situation, Background, Actions [commonly Assessment], Requests, Recommendations. The tool and its development are published elsewhere.^15^

### Phase 2 intervention implementation

Phase 2 involved the implementation of the 3 intervention components. The leadership coaching and the education sessions were led by the research team’s skilled facilitators, who are interprofessional educators, and assisted by the study PI. The leadership coaching involved leaders from the LTC homes, and the 1-day education sessions included both invited team members and the leaders. Huddles involved huddle training for team members and leaders who attended the education session, as well as resident and family dyads.

#### Leadership coaching sessions

The leadership coaching sessions were initiated prior to the 1-day education session and huddle implementation. The aim of the first leadership coaching session was to meet with the Director of Care, Assistant Director of Care, and managers at the LTC homes to discuss organizational priorities and quality improvement goals to engage residents and families in care, and how huddles could align with the organization’s prioritized goals and Quality Improvement Plans. Additional leadership coaching sessions were planned to build leader capacity to support the huddle process. A knowledge implementation tool—the Performance Improvement Observation Chart was utilized during the coaching sessions. The tool provided direction for leaders to identify and address enablers and barriers to change, emphasize team strengths and relationships during the process, and leverage success stories to build momentum toward subsequent successes. The leadership coaching methodology used was a combination of goal-directed and relational processes, which evolved into an evidence-based relational leadership coaching methodology.^36^

#### Education session

The goal of the 1-day education session at each LTC home was to build communication and collaborative care planning skills using the *Team Essentials for Engaging Families in Care* learning module, previously developed by the Ontario Centres for Learning, Research, and Innovation in LTC at Baycrest.^37^ One education session was delivered in-person at each LTC home for team members and leaders (from the coaching sessions) to support communication and collaboration among team members, residents, and family. The education session was based on several communication competencies using tools such as ARC (Acknowledge, Reassure, Clarify) for compassionate communication and SBAR for collaborative communication.^38^ The education session included ways to engage families in care, covering areas such as risk assessment, team communication, and compassionate responsiveness.^38^ The interactive team-based learning included simulation-based videos, small group case scenario discussions, and role-play of family-team interactions.

#### Resident- and family-led huddles

Huddles were implemented at each LTC home following initiation of the leadership coaching and completion of the education session. Huddle training was provided for team members and leaders who attended the education session, as well as resident and family dyad participants. The training included an overview of the huddle communication tool, a huddle video simulation, followed by a practice huddle using the communication tool. For the huddle video, 2 volunteer participants from the SAGE (Simulation Activities in Gerontological Education) program simulated the role of a resident and spouse care partner, and, along with the research team, demonstrated a simulated resident- and family-led huddle. A practice huddle was scheduled with each dyad using their own topic for discussion and included team members and a huddle coach to replicate the huddle process. During the study, due to the COVID-19 pandemic, the huddle training and intervention became virtual. Resident and family dyads completed the huddle training, including a practice huddle via Zoom with a research study team member and with assistance from a huddle coach. An eLearning module was developed and shared with team participants that included content from the education session and huddle training. We also developed an eLearning module for families to support virtual (distanced) care conversations with LTC home team members during the pandemic.

**Figure 2.**
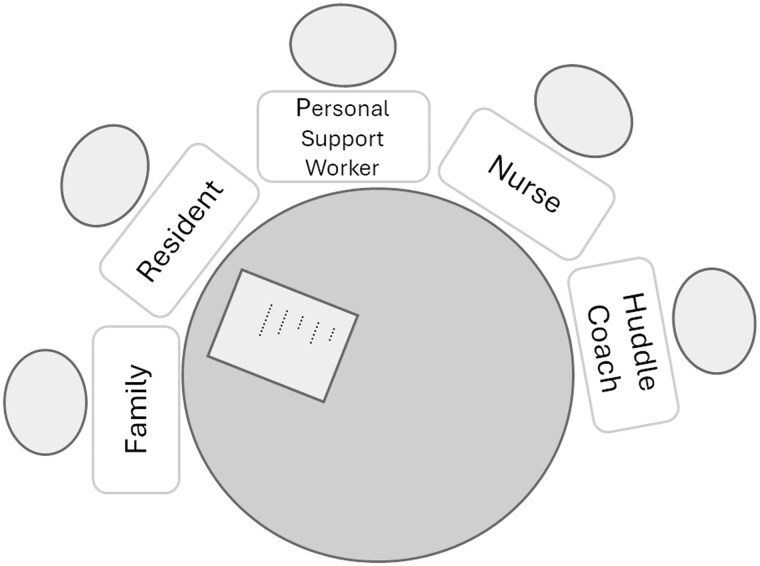
Huddle participants.

Huddles were scheduled by the huddle coach in advance based on an agreed-upon time with the resident and family. The huddles could focus on either a concern or a positive experience about care to discuss with the LTC team, based on a relationship-centered appreciative inquiry approach.^39^ Huddles were intended to initiate a collaborative process with actions for follow-up and a report back to the resident and family. To ensure feasibility in implementing the huddles, the research team aimed for each resident-family dyad to complete at least 3 huddles—approximately one per month. Resident-family dyads were asked to determine a huddle topic in advance of their scheduled huddle and review the huddle communication tool questions beforehand with their topic in mind. For each huddle, we invited 2-3 team members who had cared for the resident, had received both the education session and the huddle training, and, when feasible, whose role aligned with the topic for discussion (eg, if the topic was about morning care, then team members working the day shift would be invited to join). The huddle coach (member of the leadership team) was the timekeeper and took notes using the huddle checklist to summarize what was discussed in the huddle, including plans for follow-up (Figure 2).

### Data collection and analysis

We collected demographic data from team members, leaders, residents, and families after the practice huddle. The huddle coach completed a huddle checklist during each huddle to document the date, participants, topic discussed, actions to follow up with, and length of the huddle.

Following the completion of all huddles held during COVID-19, qualitative data were collected virtually by phone or Zoom (July 2022 to December 2022). This included a focus group with the leadership team (including the huddle coach), facilitated by the interprofessional educators and study PI. The purpose of the focus group was to evaluate the acceptability of the leadership coaching sessions and their perceptions of barriers, enablers, and successes of the huddle process, and to begin to tailor how to integrate huddles (or aspects of the huddle) into team practices. Individual interviews with huddle participants (a nurse team member, residents, and families) were conducted (maximum 30 minutes) by the PI to assess acceptability of and satisfaction with the huddles and communication tool. Participants were asked about their experience with the huddles, their perceptions of the usefulness of the huddles and the communication tool, logistics of the huddles (dose), what worked well, and suggestions for improving the huddle process and the communication tool. Individual interviews were audio-recorded and professionally transcribed verbatim. Data were analyzed using qualitative content analysis.^40^

## Results

### Leadership coaching sessions

At least one in-person leadership coaching session lasting approximately 1 hour was conducted at each of the 4 study sites prior to the COVID-19 pandemic. During the pandemic, 2 leadership coaching sessions scheduled for 1 hour were conducted virtually with the leadership team (directors, managers, social worker, and advanced practice nurse/educator) at one study site to discuss original quality improvement goals with current organizational priorities, how huddles could address these, and their experiences with the huddle process. An identified barrier to huddle conversations was that these needed to be longer than 15 minutes, and identified enablers were having the structured communication tool to guide the huddle and that huddles could support existing processes such as team rounds. An implementation success story shared by the huddle coach (leader) was that they had a “family huddle” without pulling the whole team into the brief discussion. The leader participants indicated the coaching sessions were helpful and useful to work through how huddles could address organizational goals to enhance resident participation in care and team relational skills. One leader noted: *“I like the huddle model and discussing how can we build the huddle model into usual care; we need to build in these less formal huddles because family conferences are quarterly and too long in between.”*

### Education sessions

**Figure 3.**
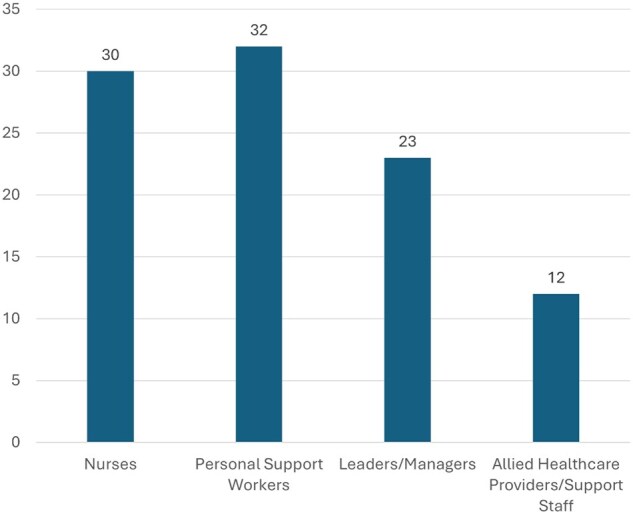
Number of team members and leaders who completed the in-person education session.

The education sessions took place between May and November 2019, with a total of 74 team members and 23 leaders (eg, managers, directors of care, administrators) participating from the 4 LTC homes (*N *= 97) (Figure 3).

#### Resident- and family-led huddles

Prior to COVID-19, 7 resident-family dyads were recruited from across the 4 LTC homes who completed the huddle training and a practice huddle. Of these, 2 dyads completed 1 huddle, and 1 dyad completed 2 huddles. Huddles were composed of the resident-family dyad, 2 to 3 team members, and a manager or social worker as huddle coach. Those who participated in the education session were invited to join a huddle, although not all participated in one. We found that huddles could be feasibly scheduled monthly (though not always consecutive months) and conducted in-person within a 15-20-minute timeframe. During COVID-19, we recruited 3 new resident-family dyads, all of whom completed a practice huddle and 3 huddles.

During the COVID-19 pandemic, 15 participants completed the study (6 leaders, 3 nurses, 3 residents, and 3 family members). These 3 resident-family dyads included a spousal relationship and children–parent relationships. Resident length of stay in the LTC home ranged from 4 to 7 years. Huddles were scheduled by the huddle coach and held during the day shift. Huddles were conducted in a private meeting room at the LTC home. The huddles were attended by 1 to 2 nurses, a manager (on occasion, but not as a huddle coach), and a social worker, consistently as the huddle coach in each huddle. The research team joined as observers via Zoom, as did the family on 2 occasions. Compared to before COVID-19, fewer team members participated in each huddle (1-2 nurses), and most huddles were longer in duration (ie, 20-25 minutes). A resident, nurse, and huddle coach indicated that sometimes the huddle became more than one topic; the resident noted that some learning was involved, indicating it was “*a process*.*”* Huddle topics discussed included pain management, falls prevention, the impact of COVID-19 on social activities, and meals/diet.

We report the findings from the post-huddle interviews and the leadership team focus group as follows: (1) acceptability of the huddles; (2) usefulness of and satisfaction with the communication tool; and (3) envisioning future uses to inform huddle innovation. Additional illustrative quotes are provided in Table 1.

**Table 1.** Additional illustrative quotes from the post-huddle interviews and leadership team focus group.

Theme	Illustrative quotes
**1. Acceptability of the huddles**	
**Feeling listened to and having a voice**	*“She [huddle coach] was taking notes and was very thoughtful in repeating what we had said that validated we were being listened to.”* (Family member - Daughter) *“I thought they were a good idea because they actually scheduled the opportunity to discuss something; … I think it was a good experience all around. I think it gave mom a voice which is important… she brought it up [the issue], I wasn’t aware of that. So it was great that she was able to articulate that and bring it up… they’ve taken her suggestions and acted on them, which is of course, the most important part of the whole thing.”* (Family member - Daughter)
**Huddling for small issues and positive experiences**	*“It can be useful for simple practical problems…to resolve an issue before it gets to be a real problem.”* (Family member - Spouse) *“… you want to include the positive experiences as well, so people focus on both.”* (Family member - Daughter) *“It’s better to hear little things as they arise to build trust when bigger problems happen and that require a greater investment.”* (Huddle Coach)
**Supporting person- and relationship-centered care**	*“Huddles can help build relationships, so you are able to advocate for residents, it’s a co-advocacy model.”* (Director of Care)
**Refining the huddles**	*“I Zoomed in once; it does ease the distance, and I do understand that it’s a great way for everyone to be in the room but it’s still not the same as a group together in a room.”* (Family member - Daughter) *“It’s probably easier in-person you can see people’s reaction that way… I can see if somebody changes their expression…”* (Resident 2)
**2. Usefulness of and satisfaction with the communication tool**	
**Prioritizing a huddle topic for discussion**	*“I think the huddles were actually more helpful than either one of us ever expected.”* (Family member - Daughter) *“The tool was helpful for our discussion to help us express is this a topic we want to talk about; it helped us prioritize what we felt was a good topic.”* (Family member - Daughter)
**Guiding a huddle care conversation**	*“I think it’s a good way to focus the conversation.”* (Family member - Daughter) *“You know, it’s one thing to say, this is not working too well as opposed to, what are we going to do about it?”* (Family member - Spouse)
**Post-huddle debrief**	*“…and for the staff as well, you’re satisfied because something happened, like, you know, something positive happened with that discussion.”* (Nurse)
**3. Envisioning future uses to inform huddle innovation**	*“I would recommend it [huddles] for all new arrivals to the home.”* (Resident 1) *“We could make a note on the chart that we huddled, and this is what came from it.”* (Huddle Coach)

#### Acceptability of the huddles

Huddle participants provided their perspectives on the acceptability of huddles in terms of appropriateness, suitability, and usefulness, including their process and format. Four themes were: (a) feeling listened to and having a voice; (b) huddling for small issues and positive experiences; (c) supporting person- and relationship-centered care; and (d) refining the huddles.

##### Feeling listened to and having a voice

Residents and family indicated that they felt listened to and enjoyed the huddles. *One resident commented: “I liked the unique concept, I can’t think of anything comparable or situation similar to this, I enjoyed it”* (Resident 3). The dyads found huddles and the communication tool useful to lead a huddle to communicate care needs. One resident stated: *“I was able to express my views and opinions on certain things… they [huddle coach] were asking my opinion, which is a positive in itself, so I would express it and my wife from her point of view—that in itself is beneficial”* (Resident 1). A family member commented on the collaboration afforded by the huddle: *“It was a nice opportunity to voice our opinions in a collaborative effort…communication is fundamental to get things changed; you need to have things voiced”* (Spouse). The nurse described how huddles created some time to sit and talk with residents and family about resident care: *“… we used to have time to talk to the residents, ask for their concerns…but not lately.”*

##### Huddling for small issues and positive experiences

Huddles were found useful for discussing smaller, practical concerns as well as positive aspects of care; a family member noted: “*I think it’s an opportunity to talk about things that aren’t working for you and also to talk about things that are working*” (Spouse). A member of the leadership team also found huddles useful to address smaller issues.

##### Supporting person- and relationship-centered care

The nurse described how the huddles and communication tool were helpful to better understand residents’ needs and communicate their needs to the team. The nurse further highlighted the importance of family presence in huddles to support the resident. A member of the leadership team also described huddles as an opportunity to support person- and relationship-centered care by *“understanding key parts of what’s important to a resident and family member”* (Director of Care). Leaders described huddles as: (1) a strategy to build trusting relationships with residents and family; (2) an informal check-in tool; and (3) a co-advocacy model.

##### Refining the huddles

Participants were satisfied with the huddles. When asked for areas for improvement, some huddle participants noted areas where the huddle process (timing) could be improved. Although the family described how the 15-minute timeframe for the huddles was an *“easy commitment,”* most huddles during the pandemic took 20-25 minutes. Huddle participants, including the huddle coach, indicated that more time was needed; 20 minutes was suggested overall. One resident mentioned that it would be helpful to have more team members in the huddle. Prior to COVID-19, the huddles had more team members participating; however, during the pandemic, fewer team members took part. A family member suggested that having the huddles every 6 weeks, rather than monthly, may help with team participation.

Although some families found the Zoom option *“convenient”* and a *“good alternative”* to traveling to the LTC home, residents and families both preferred in-person huddles.

#### Usefulness of and satisfaction with the communication tool

Huddle participants described the communication tool was useful at 3 timepoints: (1) pre-huddle to prioritize the huddle topic for discussion; (2) during the huddle to stay focused on the topic and guide the discussion; and (3) immediately post-huddle to summarize the discussion and identify actions for follow-up.

##### Prioritizing a huddle topic for discussion

Residents and family indicated that the communication tool was helpful in organizing their thoughts ahead of time and prioritizing a topic for huddle discussion. A family member further discussed how they used the tool to prioritize a topic pre-huddle: *“I think that my father [resident] and I both enjoyed the experience of a huddle. Not just the huddle itself, but the pre-huddle. So, our conversations with what topic you’d like to present at the huddle, and it made us think differently than we normally would.”*

##### Guiding a huddle care conversation

Huddle participants found the communication tool was useful for guiding the conversation during the huddle, staying focused on the topic, and considering what could be done about the situation/topic discussed. One resident stated: *“…focusing on one topic was a good point of it all.”* (Resident 1). A family member noted: *“The communication tool was good because it’s a simple way of allowing people to collect their thoughts…”* (Daughter). Although sometimes more than one topic was discussed during a huddle, residents and family described it as a learning process.

##### Post-huddle debrief

The communication tool and huddle checklist served as a huddle debrief tool whereby the huddle coach had taken notes to summarize the discussion and identify actions for follow-up, and by whom. This process facilitated outlining a plan with the team and a report back to the resident and family. The nurse described the importance of follow-up after the huddle to ensure there is an outcome: *“It’s important to know the outcome, that there is a positive outcome from out of that huddle discussion, right? So that’s a good feeling for everyone…It’s not like, oh, we discussed it and nothing happened.”* Huddle participants indicated that they were satisfied with using the communication tool. One family member suggested having an electronic version with fillable boxes as an additional option to the paper version.

#### Envisioning future uses to inform huddle innovation

Leaders described huddles as a process to be combined with an existing formal work structure going forward; for example, implement resident- and family-led huddles between quarterly family conferences; resident- and family-led huddles could complement team rounds, or during nurses’ completion of quarterly resident assessments (using the Resident Assessment Instrument-Minimum Data Set [RAI-MDS]). Resident and family recommended keeping the huddles scheduled and thereby formal; for example, a family member noted: *“I think you still have to do the formal part; schedule it depending on who you wanted to participate”* (Daughter). Some residents and family noted that huddles would be useful for residents new to the LTC home and for family who lived far from the LTC home.

## Discussion and implications

The aim of this study was to assess the feasibility, acceptability, and satisfaction of an intervention that engaged LTC home residents, their family caregivers, and the care team in a collaborative care planning process to support person- and relationship-centered care. The novel aspect of our study was that huddles were co-led by the resident and their family caregiver, focusing on either a concern or a positive experience. This approach supported person-centered care planning and decision-making tailored to the resident’s needs, values, and preferences.^29^^,^^30^ The intervention was feasible to implement in LTC homes, particularly with support from interprofessional educators who helped facilitate the leadership coaching and education sessions, and the huddle training.

During the study, we found that 3 leadership coaching sessions were feasible to complete. Huddles were conducted in approximately 20 minutes, and 2 to 3 team members and a huddle coach were a feasible number to include in each huddle. Based on the study findings, future huddles could be planned monthly or every few months. Huddles and the leadership coaching sessions could be adapted virtually.

Participants considered the huddles acceptable and reported being satisfied with them, and they found the communication tool useful. Studies evaluating the feasibility of care team huddles implemented in LTC homes have shown that they are feasible and acceptable to participants.^25^^,^^41^ In this study, participants made suggestions to further innovate huddles (e,g., to complement team rounds). Leaders enjoyed the leadership coaching sessions and found them useful to plan for quality improvement.

### Acceptability of the huddles

Huddles offer a structure and process for care planning that is appreciative and builds trusting relationships for the co-production of care. Although a huddle is not a complex intervention, investment in training and time commitment is required for participants. This includes leadership coaching sessions, an education session for team members and leaders on communication skills, and training on how to lead (dyad), facilitate (leaders), and participate in a huddle using a video simulation, education on communication tool use, and doing a practice huddle. LTC homes often face time constraints, primarily due to staffing shortages, which lead to increased workloads and limit the amount of time team members can spend with residents.^34^ Huddles can provide dedicated time to discuss resident care outside of the formal care conferences. Recent research supports our findings of the usefulness of huddles to support care coordination and communication.^19^^,^^24^ A key component of the huddles was an action plan and follow-up process with the appropriate team members to provide timely updates to residents and families. In LTC, post-fall huddles have been used with residents to develop action plans for care.^42^

### Usefulness of and satisfaction with the communication tool

Huddle participants described the communication tool as most useful at 3 timepoints: (1) pre-huddle, particularly to prioritize a topic to focus the discussion; (2) during the huddle to focus the discussion; and (3) post-huddle as a debriefing tool. Previous research has reported the usefulness and effectiveness of communication tools to guide huddle discussions in LTC^25^ and in other healthcare settings.^19^ For example, a huddle toolkit for nurse practitioner-led huddles in LTC, including a team communication guide, effectively improved communication and practice among team members.^25^ Additionally, communication skills training within LTC homes has been shown to enhance team communication^37^ and collaborative decision-making among team members, residents, and family.^11^

### Envisioning future uses to inform huddle innovation

Feedback on the acceptability and satisfaction with the huddles provides opportunities to further innovate and co-design their structure, process, and potential uses in future studies. Huddles could be considered an innovation compatible with routine practices. The leadership team described how huddles could be integrated into existing practices, such as team rounds or documentation procedures, to support care coordination. Use of a huddle checklist and a Huddle Observation Tool^19^^,^^24^ could help standardize the huddle process. Huddles support meaningful engagement of residents and a relationship-centered approach to care where families are integrated into the team as essential care partners, further legitimizing their important role and contributions within LTC homes^10^ and challenging the rhetoric of family as “visitors.”^43^ Huddles supported shared decision-making by promoting collaborative communication among all participants, helping to build shared knowledge, mutual trust, and understanding.^29^^,^^30^

Although in-person huddles were preferred, we also found that a virtual huddle was feasible and convenient for the family at times (eg, when they could not travel to the LTC home). The huddle communication tool could be helpful to support long-distance caregiving. Long-distance caregivers are a growing demographic of caregivers who provide care from a geographic distance.^44^ Long-distance caregivers perceive geographical distance as a communication barrier and may benefit from novel interventions such as communication technologies.^44^ Falzarano and colleagues (2022)^44^ found that long-distance caregivers were less satisfied with communication and information received from LTC team members than those interacting with healthcare providers while caring for an older adult in the community.

Our findings support other formats of the paper huddle communication tool, including an eLearning module and the potential for an online fillable format. Gaur and colleagues (2020)^45^ developed a communication and care planning tool to provide a structured approach to advanced care planning conversations with residents and family and to support shared decision-making remotely (phone or videoconference), specifically concerning COVID-19 infections. A digital application (app) is another option. Indeed, since the onset of COVID-19, promising practices and innovations have been explored in LTC, such as digital technologies to support person-centered care.^46^ Mobile apps have been developed to support communication in LTC; however, they have limited person-centered features and lack co-design.^47^

### Limitations

Our study has important limitations to note. We recruited large, urban LTC homes, which may limit the generalizability of the findings to smaller or rural LTC homes. Three of the 4 LTC homes were owned by for-profit organizations. Canadian studies comparing various ownership models indicate that for-profit LTC homes have lower staffing levels/fewer hours of care per resident per day than not-for-profit homes.^48^^,^^49^ However, the Ontario Government has recently set a target for nurses and personal support workers to provide an average of 3 hours and 42 minutes of direct care per resident per day by March 31, 2024.^6^ We included residents who had a local substitute decision-maker and excluded those with severe cognitive impairment. Additionally, COVID-19 interrupted our ability to continue with the huddles or to further recruit new resident-family dyads at some LTC homes, resulting in a small sample size, which limits generalizability. It was particularly difficult to recruit families when they could not enter the LTC homes; however, this led to the development of the eLearning module for family and virtual huddle training.

This study reports findings from a small feasibility study. Additional pilot testing is needed to determine the optimal huddle dose and to further evaluate both the huddle process and the communication tool. Further research should also examine the impact of huddles on work processes and resident and team member outcomes. Future implementation studies are needed to examine the effectiveness of huddles to facilitate their adoption into routine practice.^19^ A significant gap remains in evaluating the use of huddles in the LTC sector that incorporates input from residents, family care partners, and other nonclinical team members.^19^ Such studies would strengthen the evidence base for best practices for engaging LTC residents and their families as care partners in meaningful activities.^17^

### Conclusions

Our study has shown that huddles provided both a structured approach and a relational process that facilitated communication among team members, residents, and families. Although huddles require an investment in participant training and time, they can support meaningful engagement of residents and families as active partners in the co-production of care. Huddles can also strengthen resident advocacy. The communication tool provided a common language for discussing resident care. Huddles were feasible to implement and adaptable for virtual delivery. Participants viewed them favorably and considered them acceptable. Further testing of the huddle process and communication tool is needed in LTC settings and for potential use by long-distance caregivers. The communication tool could be further co-developed, including as a digital app, to support person- and relationship-centered care.

## Data Availability

The data that support the findings of this study are not publicly available due to ethical restrictions. The study was preregistered with ClinicalTrials.gov - NCT04026698.
